# Differences in brachial and femoral artery responses to prolonged sitting

**DOI:** 10.1186/1476-7120-12-50

**Published:** 2014-12-15

**Authors:** Saurabh S Thosar, Sylvanna L Bielko, Chad C Wiggins, Janet P Wallace

**Affiliations:** Department of Kinesiology, Indiana University School of Public Health, Indiana University, Bloomington, Indiana USA; Oregon Health and Science University, 3181 SW Sam Jackson Park Road, L 606/RJH 1553, Portland, OR 97239 USA

**Keywords:** Sedentary, Flow mediated dilation, Prolonged sitting, Limb differences

## Abstract

**Introduction:**

It is unknown if there are limb differences in vascular function during prolonged sitting.

**Purpose:**

This study was designed to test whether the effects of prolonged sitting on brachial artery (BA) and the superficial femoral artery (SFA) are similar.

**Methods:**

Twelve men (24.2 ± 4 yrs.) participated in a 3 hr prolonged sitting trial (SIT). SFA and BA flow mediated dilation (FMD) and respective flow patterns were measured at baseline, 1 hr, 2 hr and 3 hr.

**Results:**

By a one-way ANOVA there was a significant decline in SFA FMD during 3 hrs of SIT (p < 0.001). Simultaneously, there was a significant decline in antegrade (p = 0.04) and mean (0.037) shear rates. By a one way ANOVA there were no significant differences in BA FMD during 3 hrs of sitting. There were no changes in the shear rates in the BA except for a significant decrease in antegrade shear rate (p = 0.029) and a significant increase in oscillatory shear index (p = 0.034) during 3 hrs of sitting. Furthermore, there was no correlation between BA and SFA FMD measurements.

**Conclusion:**

Three hours of sitting resulted in impaired SFA FMD but not BA FMD. Although 3 hours of sitting did not impair BA FMD, it impaired shear patterns in the BA.

## Introduction

The presence of sedentary behavior is a major concern in today’s society [1]. There are numerous sitting opportunities throughout the day and the average sitting time for American adults is ~8 hr/day [2]. Sitting creates a unique anatomical and physiological milieu in the legs including bends in the femoral and popliteal artery, increased viscosity [3], blood pressure [4] and decrease in arterial shear rates [5]. Interestingly in humans, lower extremities exhibit an increased distribution of atherosclerosis as compared to the upper extremities [6–8]. There may be various reasons for this disparity. For example, one of the mechanisms may be increased hydrostatic pressure [4] on the lower extremities on assuming an upright posture while seating or standing. Because of the increased presence of sitting, we recently studied the effects of 3 hours of sitting on lower extremity (superficial femoral artery [SFA]) endothelial function and discovered that 3 hrs of prolonged sitting decreased mean and antegrade shear rates in the SFA and also impaired endothelial function measured by flow mediated dilation (FMD) [5]. It is known that decrease in antegrade shear rate may promote a pro-atherogenic environment [9]. In addition, FMD is a very good predictor of cardiovascular disease and is impaired even before the presence of frank atherosclerosis [10]. Nitric oxide which is a key regulator of endothelial function is synthesized in the presence of enzymes known as nitric oxide synthases. Low shear stresses during prolonged sitting [5] decrease the expression of nitric oxide synthase [11] and this may also lead to impairment in endothelial function, putting the vasculature at risk of atherosclerosis. It is interesting to note that increases in shear stresses leads to betterment in endothelial function [12]. In fact lower extremity exercise also improves brachial artery endothelial function which may explain the antiatherogenic effect of exercise [13] However, at the lower end of the physical activity continuum, it is unknown if lower extremity inactivity affects upper extremity endothelial function. In the presence of our recent findings on the impairment of endothelial function on sitting [5], it is interesting to study whether upper extremity (brachial artery [BA]) endothelial function is also affected on prolonged sitting. The purpose of this study was to investigate if the effects of prolonged sitting on BA and SFA are similar.

## Methods

### Study design

This study consisted of two screening visits and a sitting trial involving 3 hrs of uninterrupted sitting (SIT). FMD in both BA and SFA was measured at baseline, 1 hr, 2 hr and 3 hrs in the seated position. All procedures for the study were approved by Indiana University Institutional Review Board, and participants gave written informed consent for their participation.

### Participants

To be included, participants had to self-report that they were nonsmokers, and not taking any anti-hypertensive, lipid lowering, or anti-diabetic medications. To be included, they needed to have total cholesterol ≤240 mg.dl^-1^, triglycerides ≤200 mg.dl^-1^ and fasting blood glucose <120 mg^.^dl. We recruited individuals who performed <150 min^.^week^-1^ of moderate intensity physical activity or <75 min^.^week^-1^ of vigorous intensity physical activity [14]. Participating individuals were asked to maintain their regular diet patterns throughout the study duration and to discontinue any over the counter supplements at least 7 days prior to the sitting trial.

### Screening visits

Screening and testing visit procedures are also detailed elsewhere [5]. Briefly at the first visit, all experimental procedures were explained to the participants and they were familiarized with the lab setting. If the participants volunteered to participate in the study, a written informed consent was obtained. Height, weight and blood pressure (measured in the seated position in triplicate) were measured using standard procedures and a medical health history and habits questionnaire [5] was completed to screen for any preexisting cardiovascular or metabolic condition and physical activity levels. Blood pressure was measured during an additional screening visit, at the same time of day, also in the seated position and in triplicate to ensure that subjects were normotensive.

### Testing trial

Participants arrived at the laboratory (dark, quiet, climate controlled at 22-25°C) after an overnight fast, of at least 6 hours, between 0700 and 0900 hours. They were asked to refrain from caffeine for at least 8 hours before reporting to the lab. Once in the lab, participants remained seated without moving their legs or feet for the entirety of the testing session. They sat in a firmly cushioned chair with backrest with their legs perpendicular to the floor and feet flat on the ground. They were allowed to move their arms, for example to use a computer or do light reading which was not emotionally stressful during the non-testing periods of the trial. Participants were instructed to not perform any vigorous movements using the upper arms. Arm movement was not quantified. BA and SFA FMD (one followed by another, order alternated every measurement) and other vascular parameters were measured at baseline, 1 hr, 2 hr and 3 hr. All measurements were conducted in the seated posture. The participants did not change their posture during the entire duration of the trial.

### SFA and BA FMD

SFA and BA FMD were measured in accordance with current guidelines [15]. We chose SFA as the lower extremity vessel for its accessible location and NO mediated FMD [16]. Each measurement was performed in a dark, quiet and climate controlled (22-25°C) room. For the SFA, a 5 × 84 cm automatic blood pressure cuff (E-20 rapid cuff inflator; D.E. Hokanson, Bellevue, Wash., USA) was placed on their right thigh about 7 cm above the knee joint, distal to the site of ultrasound capture. For the BA, the automatic blood pressure cuff was placed on the right forearm as recommended in the guidelines. Images of the SFA and BA were obtained with a 2-D high-resolution ultrasound system (Terason t3000, Teratec h Corp., Burlington, Mass., USA), using a 5- to 12-MHz multifrequency linear-array transducer. Once satisfactory images of near and far arterial walls were obtained, the transducer was secured and stabilized in a stereotactic clamp, and landmarks were made on the participant's skin to ensure similar placement of the transducer for subsequent FMD procedures and shear rate assessments. In addition to imaging the arterial dimensions, Doppler ultrasound was used to concurrently measure SFA blood velocity. Doppler flow signals were corrected at an insonation angle of 60°, and the sample volume was placed in the middle of the artery.

Diameter images and Doppler measurements of blood velocity were continuously recorded for 45 s at baseline prior to cuff inflation. The automatic blood pressure cuff was then rapidly inflated to 250 mmHg and maintained for 5 min until cuff deflation. Diameter and blood velocity recordings resumed prior to cuff deflation and continued for 5 min for SFA and 3 min for the BA after deflation. Ultrasound images were continuously recorded at 5 frames·s^-1^ with Camtasia (TechSmith, Okemos, Mich., USA), and stored as .avi files [5, 8]. This procedure was repeated hourly across the sitting intervals.

Arterial diameters and blood velocities: Off-line analysis of diameters were performed using automated edge-detection software (Brachial Analyzer, Medical Imaging Applications LLC, Coralville, IA, USA) as previously described [8, 9]. This software allows the technician to determine a region of interest where the near and far vessel walls are most clear. The vessel wall borders are then detected using an optimal graph search-based segmentation that uses a combination of pixel density and image gradient as an objective function. All analyzed images were reviewed by the technician and edited when needed to ensure that diameter measures were always determined from the intima-lumen interface at the near and far vessel wall. Blood velocities were determined using custom made software selecting a region of interest that surrounded the Doppler wave. The velocity–time integral was used to calculate the mean blood velocity. The peak dilation after cuff deflation was determined using the highest 3 s moving average and was presented as a percentage change from baseline diameter (FMD%). SFA and BA shear rate used as an estimate of arterial shear stress and was calculated for each FMD% at baseline and during the post occlusion period using the following formula: Vm·D^-1^, where Vm is mean blood velocity (cm·s^-1^) and D is mean arterial diameter (cm). The oscillatory shear index (OSI) was calculated for each SR assessment as follows: |retrograde SR|/(|retrograde SR| + |antegrade SR|) [17]. Shear rate area under the curve (SRauc) was calculated as the area from the time of deflation up until peak diameter. All measurements and analysis were performed by a single researcher (ST) who was blinded to the participant identity and code of each image file.

### Statistical analysis

Descriptive analysis was performed to summarize participant characteristics. We were interested in looking at the effects of prolonged sitting individually on BA and SFA FMD and blood flow parameters. Within both arterial measurements (BA and SFA), one way ANOVA was conducted on the baseline diameter as the dependent variable. Further, within each arterial measurement sequence, a one way ANOVA was conducted on the dependent variables FMD%, antegrade, retrograde and mean shear rate, SRauc and OSI. When an effect was found, pairwise comparisons were used to locate significant differences across time (compared to baseline) in these variables. Observed effect size was reported for ANOVA interactions as partial eta squared (η^2^). Finally, a bivariate correlation was tested between BA and SFA FMD. The alpha level for statistical significance was set *a priori* at 0.05. All statistical calculations were performed using IBM SPSS Statistics 22.0 software (IBM SPSS Inc.).

## Results

We recruited and tested 12 participants, who comprised a homogenous group of apparently healthy inactive young men (Table 1).

**Table 1:** **Subject demographics**

Variable	Value
N	12
Age (yrs.)	24.2 ± 4
BMI (kg.m^2^)	23.7 ± 3.3
Systolic blood pressure (mmHg)	116 ± 9
Diastolic blood pressure (mmHg)	77 ± 6
Total cholesterol (mg/dl)	171 ± 31.2
LDL cholesterol (mg/dl)	98.2 ± 28.2
HDL cholesterol (mg/dl)	57.1 ± 13
VLDL cholesterol (mg/dl)	15.7 ± 9
Triglycerides (mg/dl)	77.4 ± 47
Glucose (mg/dl)	91 ± 6

Note: Data represented as mean ± SD.

Baseline diameters in BA and SFA: Within each artery, there was no significant difference in baseline diameters from baseline measurement (0 hr) to 3 hrs; BA (p = 0.805, η^2^ = 0.029), SFA (p = 0.263, η^2^ = 0.112).

### SFA measurements

There was a significant reduction in FMD across time in SFA each hour, from baseline to 3 hrs (p ≤ 0.001, η^2^ = 0.481). The FMD was significantly reduced at 1 hr, and remained attenuated at 2 hrs and 3 hrs. Antegrade shear rate significantly declined from baseline to 3 hrs (p = 0.049, η^2^ = 0.209). The antegrade shear rate was significantly lower at 1 hr and 2 hr but not at 3 hr as compared to baseline. There was a significant decline in mean shear rate from baseline across time (p = 0.028, η^2^ = 0.237). Mean shear rate was significantly lower at 1 hr and 2 hr but not at 3 hr as compared to baseline. Retrograde shear rate was not significantly different from baseline (p = 0.940, η^2^ = 0.012). OSI also did not change from baseline to 3 hours (p = 0.107, η^2^ = 0.116). SRauc did not show any significant change from baseline (p = 0.054, η^2^ = 0.459) [SRauc data are for 5 participants (please see the discussion section)]. In summary, in the SFA, there was a significant decline in antegrade shear rate, mean shear rate and FMD during 3 hrs of prolonged sitting.

### BA measurements

**Figure 1:**
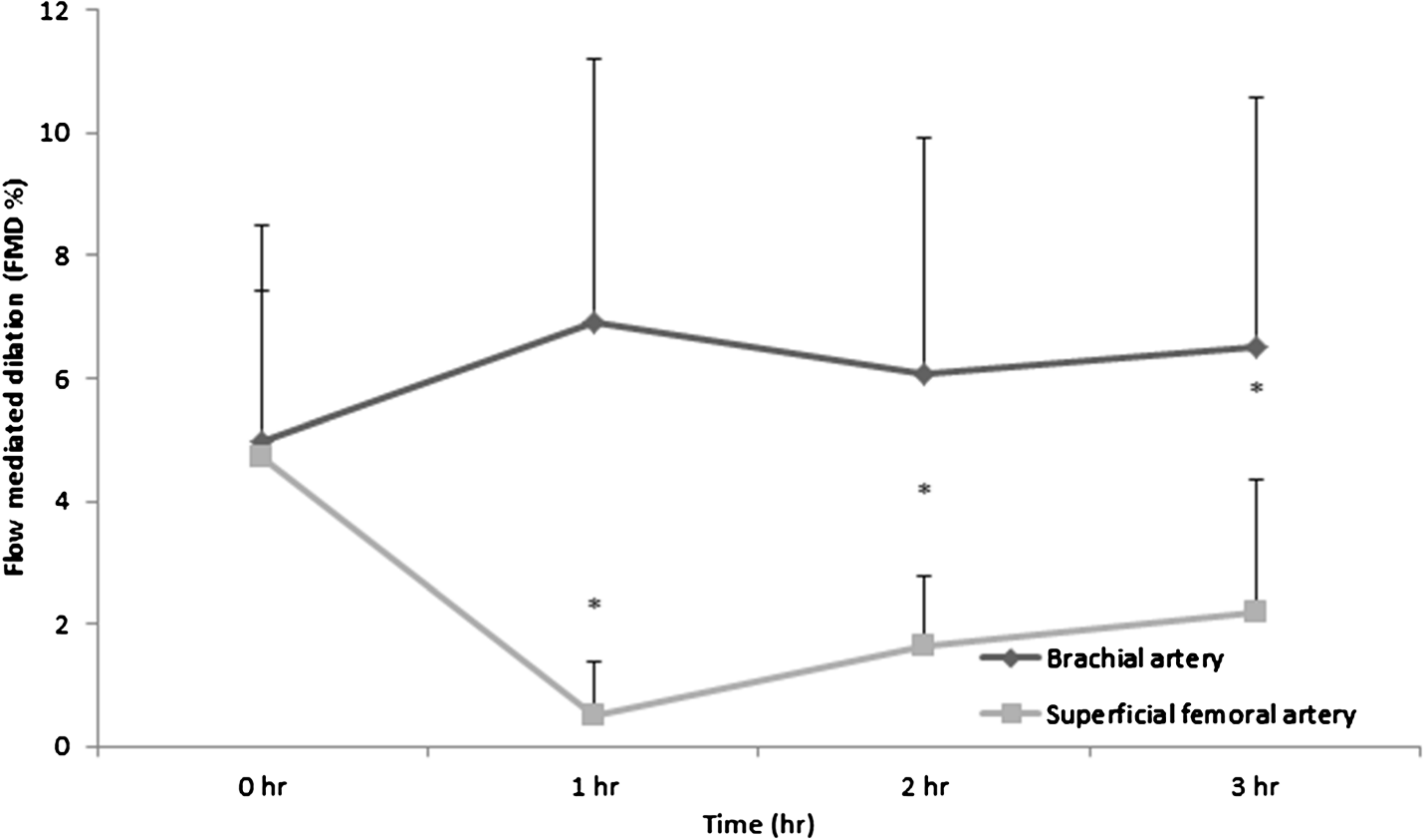
**Flow mediated dilation in the brachial and superficial femoral artery during 3 hours of sitting during the sitting.** Error bars represent standard deviations. * Indicates significant difference from baseline in the SFA at p≤0.05.

There was no significant difference in BA FMD from baseline across 3 hrs (p = 0.602, η^2^ = 0.054). There was a significant decline in the antegrade shear (p = 0.029, η^2^ = 0.236) and increase in OSI (p = 0.034, η^2^ = 0.228), but no significant changes in the retrograde shear rate (p = 0.457, η^2^ = 0.075), mean shear rate (p = 0.481, η^2^ = 0.71) and SRauc (p = 0.837, η^2^ = 0.031) from baseline. In summary, all brachial parameters in exception of antegrade shear rate and oscillatory shear index remain unchanged during 3 hours of sitting. All FMD data are presented in Figure 1 and baseline diameter, and shear rates are presented in Table 2.

**Table 2:** **Baseline diameters** (**mm**), **shear rates** (**s** ^-**1**^), **SRauc and normalized FMD in the BA and SFA during the SIT and VIT trial at baseline**, **1 hr**, **2 hr and 3 hr**

	Brachial artery (BA)				Superficial femoral artery (SFA)			
	0 hr	1 hr	2 hr	3 hr	0 hr	1 hr	2 hr	3 hr
**Baseline diameters**	4.0 ± 0.6	3.9 ± 0.6	4 ± 0.7	3.9 ± 0.4	6.3 ± 0.6	6.3 ± 0.7	6.5 ± 0.6	6.3 ± 0.7
**Antegrade SR**	23.6 ± 8.7	18.3 ± 8.3	19 ± 9.1	16.7 ± 5.4*	7.1 ± 2.1	5.4 ± 2.5*	5.5 ± 2.3*	6.1 ± 2
**Mean SR**	16.7 ± 10.4	11.3 ± 7.3	11 ± 9.1	12.9 ± 14.8	4.6 ± 2.1	2.5 ± 3*	2.8 ± 1.5*	3.4 ± 1.5
**Retrograde SR**	6.7 ± 4.6	6.9 ± 3.4	7.9 ± 3.7	8.1 ± 3.9	2.5 ± 1.1	2.8 ± 1.7	2.7 ± 1.4	2.7 ± 1.2
**OSI** (**a.u**)	0.22 ± 0.15	0.28 ± 0.12	0.31 ± 0.09	0.31 ± 0.07*	0.27 ± 0.1	0.36 ± 0.16	0.32 ± 0.08	0.30 ± 0.08
**Peak SR**	110.4 ± 44.5	107.6 ± 46.6	115.6 ± 58.5	102.9 ± 47.2	50.2 ± 67.3	19.7 ± 13.6	26.8 ± 14	22.2 ± 10.2
**SR**_**auc**_(**a.u**)	3083.7 ± 1505	3036 ± 1542.9	3039.2 ± 1755.9	2744.8 ± 1603.2	832.7 ± 232.9	449.5 ± 309.9	356.4 ± 221.1	525.3 ± 226.2
**FMD**:**SR**_**auc**_	0.002 ± 0.001	0.002 ± 0.001	0.002 ± 0.001	0.004 ± 0.004	0.005 ± 0.005	0.128 ± 0.0341	0.003 ± 0.002	0.003 ± 0.002

Note: *indicates significantly (p ≤ 0.05) different from baseline within each artery. Data presented as Mean ± SD.

There was no correlation between BA and SFA FMD during 3 hrs of sitting(r = -0.029, p = 0.847).

## Discussion

The purpose of this study was to investigate whether the effects of prolonged sitting on BA and SFA endothelial function are similar. We hypothesized that prolonged sitting will lead to decline in SFA endothelial function, but not in the BA endothelial function. Indeed, we found a decline in FMD in SFA during 3 hours of sitting (also published in [5]). We also saw a decline in antegrade and mean shear rate in the SFA. In the BA, we found no significant decline in FMD during prolonged sitting; however to our surprise we found a decline in BA antegrade shear rate along with a significant increase in OSI 3 hours of sitting. This is the first study to our knowledge which compares the physiological effects of prolonged sitting on the upper and lower extremity vasculature.

We have previously discovered and discussed that prolonged sitting led to decreases in antegrade and mean shear rates in addition to impairment in endothelial function in the SFA [5]. Padilla and colleagues [4] in a similar study showed that when subjected to a similar inactivity model, there is a decline in shear rate patterns in the popliteal artery but no significant change in the FMD. Our shear rate results are similar to these authors but the FMD results differ primarily because of the study design, as discussed elsewhere [5].

The focus of this discussion will be our novel finding which is the decline in the antegrade shear rate in the BA along with an increase in OSI during 3 hrs prolonged sitting. We did not expect any change in the vascular parameters in the BA during sitting, primarily because upper extremity movement was not controlled and subjects moved their upper extremities, although the movement was minimal. We have previously shown that minimal activity during breaking sitting time prevents the impairment in shear rates and FMD in the SFA during prolonged sitting [5]. Indeed, in this design, moving the upper extremities is similar to breaking sitting time for the lower extremities and therefore we did not expect any change in vascular parameters in the BA. There is only one study which has looked at the effects of acute inactivity on BA FMD. Padilla and colleagues [4] had their subjects’ arm hanging to mimic the increased hydrostatic load and inactivity. They showed that the shear rates decreased from baseline through 150 min which is similar to our results and a significant decline in BA FMD which is contrary to our findings. The type of inactivity in our protocol does not change the hydrostatic load in the upper extremities and mimics what most people do in sedentary jobs, for example using a computer or reading. Despite the movement in the arms, the antegrade shear rate declined and OSI increased. It is known that a decline in antegrade shear rate represents an aging profile in the vasculature [9]. It is also known that oscillatory shear affects nitric oxide production thus inducing oxidative stress [18] and monocyte adhesion [19]. Oscillatory shear patterns are also associated with increased atherosclerosis [20]. Newcomer and colleagues have shown that in the upright posture, mean blood velocity and shear rate are lower in SFA than BA [21]. In view of this result and our data, it is clear that prolonged sitting causes an impairment in the BA shear rate patterns, although the BA FMD appears to be preserved as compared to SFA FMD. There are indeed other studies which have looked at the effect of physical inactivity on upper extremity vascular function during bed rest [22], dry water immersion [23], spinal cord injury [24] among other inactivity models. However our results are directly important for public health and are shown in a study design simulating normal human behavior.

There are various possibilities why BA FMD might not have changed even in the presence of a reduced antegrade shear rate and increased OSI. It is possible that longer duration of sitting may have shown an effect on BA FMD. Antegrade shear rate declined in the SFA at 1 hr but it declined in the BA at 3 hours. This possibly shows some latency in the impairment of BA function on prolonged sitting, perhaps due to the regular movement of the upper extremities. It is possible that multiple bouts of prolonged sitting may impair BA FMD which is a marker of systemic vascular function [25]. The possibility that the decline in FMD in the SFA during sitting signifies a true local phenomenon and not a systemic phenomenon cannot be discounted. A chronic environment of impaired endothelial function may lead to the increased atherosclerosis in the legs. Indeed 2 hours of siting has been shown to increase whole blood viscosity in the legs but not the arm [3]. Furthermore, there are known differences in vascular reactivity between BA and FA [26] and hence one can speculate that the FMD in the BA may be preserved even when the FMD in SFA is blunted, chronically exposing the lower extremity to a pro-atherogenic milieu. Finally it is possible that BA FMD may not be a sensitive measure of systemic endothelial function during prolonged sitting.

### Mechanisms

There are various possible mechanisms leading to an impaired shear pattern in the BA. For example, increase in muscle sympathetic nerve activity may lead to impaired shear patterns [27]. We could not measure MSNA in our study due to logistical reasons. It is also possible that prolonged sitting may have led to an increased blood pressure [28] which further led impaired shear patterns in the BA. It is known that lower extremity exercise increases BA FMD via an increase in shear rates [29]. Based on our results, we can postulate that although 3 hours of sitting does not change BA FMD, it does promote pro-atherogenic shear patterns [9].

### Limitations

This study is not without limitations. For example, we did not measure blood pressure during the seated period. We contemplated measuring blood pressure but this was logistically difficult to do in the ipsilateral leg for concerns of changing blood flow patterns and in the contralateral leg for concerns of moving the lower extremity. It could have been done in the arm but because the primary purpose was comparing the two arteries it was not measured in the BA as well. It is known that uninterrupted sitting is associated with increased resting BP albeit in overweight adults [30] which could lead to impaired shear rates and endothelial function. We acknowledge that not having blood pressure measures is a mechanistic limitation. We did not measure viscosity. It is known that whole blood viscosity increases in the leg but not in the arm during 2 hours of sitting. Our design consisted of 3 hours of prolonged sitting and it is possible that viscosity increases in the arm after 3 hours especially since increased viscosity is associated with low shear rates [31]. Furthermore, measuring viscosity would also have allowed us to measure shear stresses. We did not measure MSNA. As discussed earlier increased MSNA has been shown to impair shear rate patterns in conduit arteries [27]. Finally, we had young inactive men as our participants and more research needs to be done in women and different age groups to generalize these results to other populations.

Despite these limitations, our study adds significant new information to the existing literature on physical inactivity and the vascular differences between upper and lower extremities. This is the first study which shows that 3 hrs of sitting decreases antegrade shear rate and increases OSI in the BA, both shear patterns harmful to vascular health.

## Conclusions

3 hrs of sitting leads to significant decline in antegrade and means shear rates in SFA, also impairing FMD. In the BA, there is a significant decline in antegrade shear rate and increased OSI without a clear impairment of FMD. Prolonged sitting is common human behavior and its effects on vasculature are not limited to lower extremities. Our current data along with previously published results [5] make a case for avoiding prolonged time in a seated posture. More mechanistic research is needed to identify the various physiological changes in the BA and SFA during prolonged sitting.

## References

[1] Harrington DM, Barreira TV, Staiano AE, Katzmarzyk PT (2014). The descriptive epidemiology of sitting among US adults, NHANES 2009/2010. J Sci Med Sport.

[2] Matthews CE, Chen KY, Freedson PS, Buchowski MS, Beech BM, Pate RR, Troiano RP (2008). Amount of time spent in sedentary behaviors in the United States, 2003–2004. Am J Epidemiol.

[3] Hitosugi M, Niwa M, Takatsu A (2000). Rheologic changes in venous blood during prolonged sitting. Thromb Res.

[4] Padilla J, Sheldon RD, Sitar DM, Newcomer SC (2009). Impact of acute exposure to increased hydrostatic pressure and reduced shear rate on conduit artery endothelial function: a limb-specific response. Am J Physiol Heart Circ Physiol.

[5] Thosar SS, Bielko SL, Mather KJ, Johnston JD, Wallace JP (2014). Effect of prolonged sitting and breaks in sitting time on endothelial function. Med Sci Sports Exerc.

[6] Taylor G, Calo A (1962). Atherosclerosis of arteries of lower limbs. Br Med J.

[7] DeBakey ME, Lawrie GM, Glaeser DH (1985). Patterns of atherosclerosis and their surgical significance. Ann Surg.

[8] Allam AH, Thompson RC, Wann LS, Miyamoto MI, El-Din AE-HN, el-Maksoud GA, Soliman MA-T, Badr I, Amer HA-R, Sutherland ML (2011). Atherosclerosis in ancient Egyptian mummies: the Horus study. J Am Coll Cardiol Img.

[9] Young CN, Deo SH, Padilla J, Laughlin MH, Fadel PJ (2010). Pro-atherogenic shear rate patterns in the femoral artery of healthy older adults. Atherosclerosis.

[10] Giannotti G, Landmesser U (2007). Endothelial dysfunction as an early sign of atherosclerosis. Herz Kardiovaskuläre Erkrankungen.

[11] Malek AM, Alper SL, Izumo S (1999). Hemodynamic shear stress and its role in atherosclerosis. J Am Med Assoc.

[12] Green DJ, Maiorana A, O'Driscoll G, Taylor R (2004). Effect of exercise training on endothelium derived nitric oxide function in humans. J Physiol.

[13] Green D, Cheetham C, Mavaddat L, Watts K, Best M, Taylor R, O'Driscoll G (2002). Effect of lower limb exercise on forearm vascular function: contribution of nitric oxide. Am J Physiol Heart Circ Physiol.

[14] CDC: **How much physical activity do adults need?** http://www.cdc.gov/physicalactivity/everyone/guidelines/adults.html

[15] Thijssen DHJ, Black MA, Pyke KE, Padilla J, Atkinson G, Harris RA, Parker B, Widlansky ME, Tschakovsky ME, Green DJ (2011). Assessment of flow-mediated dilation in humans: a methodological and physiological guideline. Am J Physiol Heart Circ Physiol.

[16] Kooijman M, Thijssen D, De Groot P, Bleeker M, Van Kuppevelt H, Green D, Rongen G, Smits P, Hopman M (2008). Flow‒mediated dilatation in the superficial femoral artery is nitric oxide mediated in humans. J Physiol.

[17] Johnson BD, Mather KJ, Newcomer SC, Mickleborough TD, Wallace JP (2012). Brachial artery flow-mediated dilation following exercise with augmented oscillatory and retrograde shear rate. Cardiovasc Ultrasound.

[18] Hillsley MV, Tarbell JM (2002). Oscillatory shear alters endothelial hydraulic conductivity and nitric oxide levels. Biochem Biophys Res Commun.

[19] Hwang J, Saha A, Boo YC, Sorescu GP, McNally JS, Holland SM, Dikalov S, Giddens DP, Griendling KK, Harrison DG (2003). Oscillatory shear stress stimulates endothelial production of from p47phox-dependent nad (p) h oxidases, leading to monocyte adhesion. J Biol Chem.

[20] Ku DN, Giddens DP, Zarins CK, Glagov S (1985). Pulsatile flow and atherosclerosis in the human carotid bifurcation: positive correlation between plaque location and low oscillating shear stress. Arterioscler Thromb Vasc Biol.

[21] Newcomer S, Sauder C, Kuipers N, Laughlin M, Ray C (2008). Effects of posture on shear rates in human brachial and superficial femoral arteries. Am J Physiol Heart Circ Physiol.

[22] Hamburg NM, McMackin CJ, Huang AL, Shenouda SM, Widlansky ME, Schulz E, Gokce N, Ruderman NB, Keaney JF, Vita JA (2007). Physical inactivity rapidly induces insulin resistance and microvascular dysfunction in healthy volunteers. Arterioscler Thromb Vasc Biol.

[23] Navasiolava NM, Dignat-George F, Sabatier F, Larina IM, Demiot C, Fortrat JO, Gauquelin-Koch G, Kozlovskaya IB, Custaud MA (2010). Enforced physical inactivity increases endothelial microparticle levels in healthy volunteers. Am J Physiol Heart Circ Physiol.

[24] Bell JW, Chen D, Bahls M, Newcomer SC (2011). Evidence for greater burden of peripheral arterial disease in lower extremity arteries of spinal cord-injured individuals. Am J Physiol Heart Circ Physiol.

[25] Neunteufl T, Katzenschlager R, Hassan A, Klaar U, Schwarzacher S, Glogar D, Bauer P, Weidinger F (1997). Systemic endothelial dysfunction is related to the extent and severity of coronary artery disease. Atherosclerosis.

[26] Newcomer SC, Leuenberger UA, Hogeman CS, Handly BD, Proctor DN (2004). Different vasodilator responses of human arms and legs. J Physiol.

[27] Padilla J, Young CN, Simmons GH, Deo SH, Newcomer SC, Sullivan JP, Laughlin MH, Fadel PJ (2010). Increased muscle sympathetic nerve activity acutely alters conduit artery shear rate patterns. Am J Physiol Heart Circ Physiol.

[28] Gotshall RW, Aten LA, Yumikura S (1994). Difference in the cardiovascular response to prolonged sitting in men and women. Can J Appl Physiol.

[29] Birk GK, Dawson EA, Atkinson C, Haynes A, Cable NT, Thijssen DH, Green DJ (2012). Brachial artery adaptation to lower limb exercise training: role of shear stress. J Appl Physiol.

[30] Larsen R, Kingwell B, Sethi P, Cerin E, Owen N, Dunstan D (2014). Breaking up prolonged sitting reduces resting blood pressure in overweight/obese adults. Nutr Metab Cardiovasc Dis.

[31] Quyyumi AA, Patel RS (2010). Endothelial dysfunction and hypertension cause or effect?. Hypertension.

